# Right coronary artery encasement by metastatic cardiac lymphoma

**DOI:** 10.1007/s12471-022-01689-5

**Published:** 2022-05-03

**Authors:** S. Borges, C. Ferreira, J. I. Moreira

**Affiliations:** Cardiology Department, Centro Hospitalar de Trás os Montes e Alto Douro, Vila Real, Portugal

A 20-year-old immunocompetent woman, without prior cardiovascular disease, presented to the emergency department for progressive exertional dyspnoea. A transthoracic echocardiogram revealed a large heterogeneous mass involving great vessels, pericardium and right ventricular wall, leading to severe right ventricular systolic dysfunction (see Movie 1–3 in Electronic Supplementary Material). Cardiac magnetic resonance imaging confirmed the presence of a large infiltrating lesion reaching from the origin of the supra-aortic trunks to the great vessels and heart, particularly at the auriculoventricular groove. The right coronary artery was enveloped by the mass, without invasion or compression (Fig. 1).

**Fig. 1:**
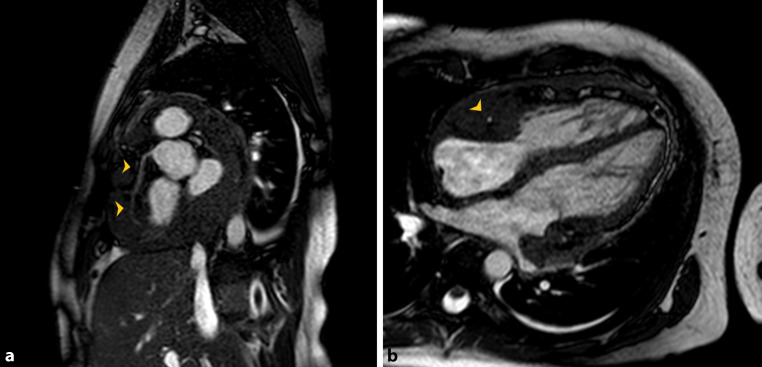
Cardiac magnetic resonance imaging—short axis view (**a**) and 4‑chamber view (**b**) showing the course of the right coronary artery inside the tumour mass (*arrow*)

This presentation was suggestive of lymphoma with massive cardiac involvement [1]. Biopsy of mesenteric adenopathy confirmed the presence of a diffuse large B‑cell lymphoma. Standard chemotherapy treatment was initiated. However, shortly after, the patient evolved to multiorgan dysfunction refractory to all resuscitation efforts and died 2 days later.

Predilection for the auriculoventricular groove and right coronary artery encasement are radiologic features that should raise suspicion of this diagnosis [2].

## Supplementary Information

Movie 1*—*Transthoracic Echocardiogram Short Axis View

Movie 2*—*Transthoracic Echocardiogram Long Axis view

Movie 3*—*Transthoracic echocardiogram*—*4 Chambers view
